# Profiling sugar metabolism during fruit development in a peach progeny with different fructose-to-glucose ratios

**DOI:** 10.1186/s12870-014-0336-x

**Published:** 2014-11-25

**Authors:** Elsa Desnoues, Yves Gibon, Valentina Baldazzi, Véronique Signoret, Michel Génard, Bénédicte Quilot-Turion

**Affiliations:** INRA, UR1052 Génétique et Amélioration des Fruits et Légumes, F-84000 Avignon, France; INRA, UR1115 Plantes et Systèmes de Culture Horticoles, F-84000 Avignon, France; INRA, UMR 1332 Biologie du Fruit et Pathologie, F33883 Villenave d’Ornon, France; Univ. Bordeaux 146 rue Léo-Saignat, F 33076 Bordeaux, Cedex France; Metabolome Facility of Bordeaux Functional Genomics Center, IBVM, Centre INRA de Bordeaux, F-33140 Villenave d’Ornon, France

**Keywords:** *Prunus persica*, Extensive profiling, Metabolites, Enzymatic capacities, Fruit quality

## Abstract

**Background:**

Fruit taste is largely affected by the concentration of soluble sugars and organic acids and non-negligibly by fructose concentration, which is the sweetest-tasting sugar. To date, many studies investigating the sugars in fruit have focused on a specific sugar or enzyme and often on a single variety, but only a few detailed studies addressing sugar metabolism both as a whole and dynamic system are available. In commercial peach fruit, sucrose is the main sugar, followed by fructose and glucose, which have similar levels. Interestingly, low fructose-to-glucose ratios have been observed in wild peach accessions. A cross between wild peach and commercial varieties offers an outstanding possibility to study fruit sugar metabolism.

**Results:**

This work provides a large dataset of sugar composition and the capacities of enzymes that are involved in sugar metabolism during peach fruit development and its genetic diversity. A large fraction of the metabolites and enzymes involved in peach sugar metabolism were assayed within a peach progeny of 106 genotypes, of which one quarter displayed a low fructose-to-glucose ratio. This profiling was performed at six stages of growth using high throughput methods. Our results permit drawing a quasi-exhaustive scheme of sugar metabolism in peach. The use of a large number of genotypes revealed a remarkable robustness of enzymatic capacities across genotypes and years, despite strong variations in sugar composition, in particular the fructose-to-glucose ratio, within the progeny. A poor correlation was also found between the enzymatic capacities and the accumulation rates of metabolites.

**Conclusions:**

These results invalidate the hypothesis of the straightforward enzymatic control of sugar concentration in peach fruit. Alternative hypotheses concerning the regulation of fructose concentration are discussed based on experimental data. This work lays the foundation for a comprehensive study of the mechanisms involved in sugar metabolism in developing fruit.

**Electronic supplementary material:**

The online version of this article (doi:10.1186/s12870-014-0336-x) contains supplementary material, which is available to authorized users.

## Background

Peach (*Prunus persica*) has a high economic value, and with the publication of its genome [1], has become the reference species for *Prunus*. The organoleptic properties of peach, as fruit in general, largely depend on the accumulated sugars and acids, and sweetness is positively correlated with the ratio of sugars and acids [2]. The ratios between the different sugars also affect the fruit taste [3,4], with fructose being the sweetest (almost twice as sweet as glucose) [5]. Knowledge of the mechanisms that are involved in sugar metabolism is therefore essential for the creation of fruit varieties that meet consumers’ expectations.

Sugar accumulation during fruit development has been studied in different species. The amount of total soluble sugars usually changes with fruit growth, peaking at maturity or ripening [6–11]. However, sugar accumulation patterns and concentrations differ between species. In most fruits, glucose and fructose form the major proportion of soluble sugars, whereas in peach, mandarin and litchi, sucrose is the predominant sugar [8,12–14]. Sorbitol, a sugar alcohol, is present in some species, especially in Rosaceae, but its concentration varies strongly between species. For example, the level of sorbitol is very high in cherry [15], exhibiting comparable levels to those of glucose and fructose. In contrast, sorbitol is only the third–most-abundant sugar before sucrose in loquat [11] and the fourth in apple [7] and peach [12]. Generally, fructose and glucose are present in equal amounts, such as in tomato [9], melon [16], grape berry [17], cherry [15] and peach [12]. However, in apple, fructose is by far the major sugar [7]. Interestingly, natural fructose-to-glucose ratio deficits are often observed in fruit of wild [3] and ornamental [12] peaches. This feature is also encountered in apricots [18] and tomato, for which a major locus that controls the fructose-to-glucose ratio has been found [19].

Extensive studies of enzymatic capacity of fruit sugar pathways are needed to improve our understanding of mechanisms involved in sugar metabolism and in the fructose-to-glucose ratio. Peach is a good candidate for such study given its highly variable fructose-to-glucose ratio (from 0.4 to 2.5 depending on the genotype [20]), which is as variable as the range found between the main commercial fruit species.

Many studies investigating sugars in peach fruit have focused on a specific sugar or enzyme and have been performed on a single variety (Additional file 1). With recent technological developments, it is now possible to perform metabolic and enzymatic profiles using large numbers of samples. However, only a few detailed studies addressing sugar metabolism both as a whole and as a dynamic system are available in the literature: Nardozza et al. [21] carried out a multilevel analysis of kiwifruit metabolism; Steinhauser et al. [22] and Biais et al. [23] performed metabolic and enzymatic profiling during tomato fruit development; and Lombardo et al. [24] reported a large metabolic profiling of peach throughout development, but these latter authors studied only a few enzymes of carbon and nitrogen metabolism. To date, very few attempts have been made to link metabolites and enzymatic capacities to understand the mechanistic relationships that regulate sugar metabolism in fruit. A recent study using a modeling approach quantified the control that is exerted by enzymes within the sucrose metabolism throughout tomato fruit development [25].

In this context, the present study intends to provide a unique, extensive picture of the developmental dynamics and genetic diversity of sugar metabolism in peach fruit. For this purpose, a large fraction of the metabolites and enzymes involved in peach sugar metabolism were assayed within a peach progeny of 106 genotypes at different times of fruit development and using high-throughput methods [26,27].

Natural diversity provides a powerful resource for uncovering key components in the regulation of metabolic networks [28,29]. Although profiling the entire progeny erases accession particularities and highlights general trends of the species, the presence within our progeny of genotypes displaying contrasting fructose-to-glucose ratios offers a useful system for exploring sugar metabolism in the case of a major perturbation in the fructose amount. Indeed, our results revealed the remarkable robustness of enzymatic capacities across genotypes and years despite strong variations in the sugar composition as well as in the fructose-to-glucose ratio within the population. A poor correlation was found between the enzymatic capacities and metabolite concentrations, discarding the hypothesis of a straightforward enzymatic control of sugar concentration in fruit. The data obtained allow the formulation of alternative hypotheses concerning the regulation of fructose concentration.

## Results

**Figure 1:**
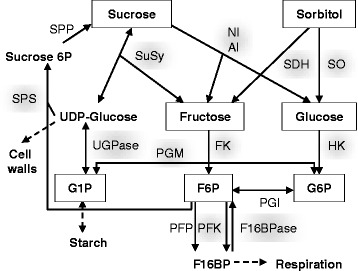
**Peach sugar metabolism [** 3 **,** 12 **,** 30 **].** Proposed sugar metabolism pathway in peach fruit as built from partial literature data. The metabolites and enzymatic capacities that were assayed in the present study are framed and highlighted. In peach, carbon enters the fruit in the form of sucrose and sorbitol, the two main end products of photosynthesis in source organs [12]. Sucrose, which is the major sugar in peach fruit, is hydrolysed into fructose and UDP-glucose by sucrose synthase (SuSy, EC 2.4.1.13) in the cytosol and into fructose and glucose by invertases, in the cell wall, the cytosol (NI, neutral invertase EC 3.2.1.26) or the vacuole (AI, acid invertase EC 3.2.1.26). Cytosolic sorbitol is converted to fructose via sorbitol dehydrogenase (SDH, EC 1.1.1.14) and to glucose via sorbitol oxidase (SO) [12]. Fructose and glucose can be stored in the vacuole or phosphorylated in the cytosol via reactions catalyzed by fructokinase (FK, EC 2.7.1.4) and hexokinase (HK, EC 2.7.1.1), respectively, to form fructose-6-phosphate (F6P) and glucose-6-phosphate (G6P) [3]. F6P is then converted into fructose 1,6-bisphosphate (F16BP) by PPi-phosphofructokinase (PFP, EC 2.7.1.90), ATP-phosphofructokinase (PFK, EC 2.7.1.11) and the inverse reaction is catalyze by fructose-1,6-bisphosphatase (F16BPase, EC 3.1.3.11) [30]. Phosphoglucose isomerase (PGI, EC 5.3.1.9), phosphoglucomutase (PGM, EC 5.4.2.2) and UDP-glucose pyrophosphorylase (UGPase, EC 2.7.7.9) catalyze the inter-conversion between the hexose phosphates. Sucrose phosphate synthase (SPS, EC 2.4.1.14) and sucrose-phosphate phosphatase (SPP EC 3.1.3.24), involve in the sucrose re synthesis via a futile cycle.

From the sparse information available in the literature, we built a comprehensive scheme of sugar metabolism in peach fruit (Figure 1) that includes all of the known enzymatic reactions as well as connections to the main pathways of carbon metabolism. Accordingly, the metabolic profiling of the 106 genotypes of an interspecific progeny was performed by assaying four metabolites and twelve enzymatic capacities (maximal activities) at different times of fruit development. In addition, the three major hexose phosphates were assayed in ten genotypes of the progeny throughout fruit development.

The large number of studied genotypes permits the blanking out of individual variations to extract the general characteristics of peach sugar metabolism. Within the large phenotypic diversity displayed by our progeny, 77 out of the 106 individuals under study exhibited a ‘standard’ fructose-to-glucose ratio. We first focused on these individuals to identify general trends of temporal variations during growth in peach fruit. In the second step of the analysis, we focused on the fructose type, comparing the ‘low-fructose-to-glucose-ratio’ genotypes to the ‘standard’ ones. The results from this new point of view may indicate the key regulatory steps of sugar metabolism.

### Temporal evolutions of metabolites and enzymatic capacities during fruit growth

**Figure 2:**
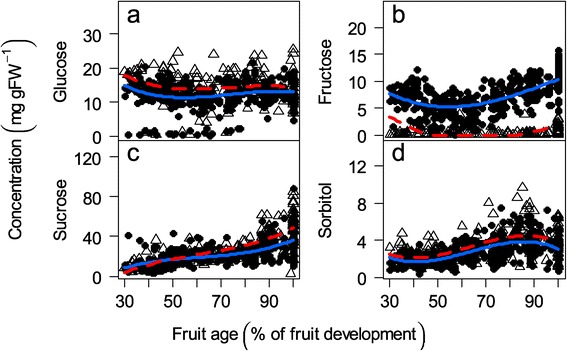
**Metabolite profiles.** Changes in the metabolite concentrations (mg g FW ^−1^) during fruit development for 106 genotypes. **a)** Glucose, **b)** Fructose, **c)** Sucrose and **d)** Sorbitol concentrations. The symbols represent the mean of the two technical replicates, open triangles represent the ‘low-fructose-to-glucose-ratio’ genotypes, and closed circles represent the ‘standard-fructose-to-glucose-ratio’ genotypes. The lines are fitted linear models by GLMM for all of the ‘low-fructose-to-glucose-ratio’ genotypes (red dashed line) and for all of the ‘standard-fructose-to-glucose-ratio’ genotypes (blue solid line).

**Figure 3:**
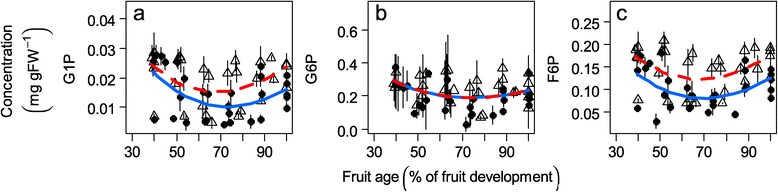
**Hexose phosphate profiles.** Changes in the hexose phosphates concentration (mg g FW ^-1^) during fruit development for ten genotypes. **a)** G1P (glucose-1-phosphate), **b)** G6P (glucose-6-phosphate) and **c)** F6P (fructose-6-phosphate) concentrations. The symbols represent the mean of the three biological and the two technical replicates, open triangles represent the ‘low-fructose-to-glucose-ratio’ genotypes and closed circles the ‘standard-fructose-to-glucose-ratio’ genotypes. The segments represent the standard error on biological replicates. The lines are fitted linear models by GLMM for all of the five ‘low-fructose-to-glucose-ratio’ genotypes (red dashed line) and for all of the five ‘standard-fructose-to-glucose-ratio’ genotypes (blue solid line).

The sucrose concentration rapidly increased with time to become the main sugar in peach fruit (Figure 2c). In contrast, the glucose concentration remained relatively constant (Figure 2a), and the fructose concentration decreased during the first part of the development and then increased (Figure 2b). The fructose and glucose amounts were comparable at the end of the time courses. Sorbitol, which was always lower than the three other sugars analyzed, displayed a slight increase during growth and then decreased during the latest developmental stages (Figure 2d). As expected, the hexose phosphates were present at considerably lower concentrations than the previous sugars. Their time courses were similar, with glucose-6-phosphate (G6P) being higher than fructose-6-phosphate (F6P) or glucose-1-phosphate (G1P) (Figure 3). The results obtained for the metabolites were in general in accordance with those from the literature (Additional file 1) in terms of magnitude and time courses. The high variability in the sugar concentration between the genotypes in our population is coherent with the ranges observed by Cantin et al. [20]. Concerning hexose phosphates, the literature on peach is poor. Kanayama et al. [3] reported F6P and G6P concentrations that were tenfold less than those that we obtained. However, our results for F6P are consistent with the results from Lombardo et al. [24].

**Figure 4:**
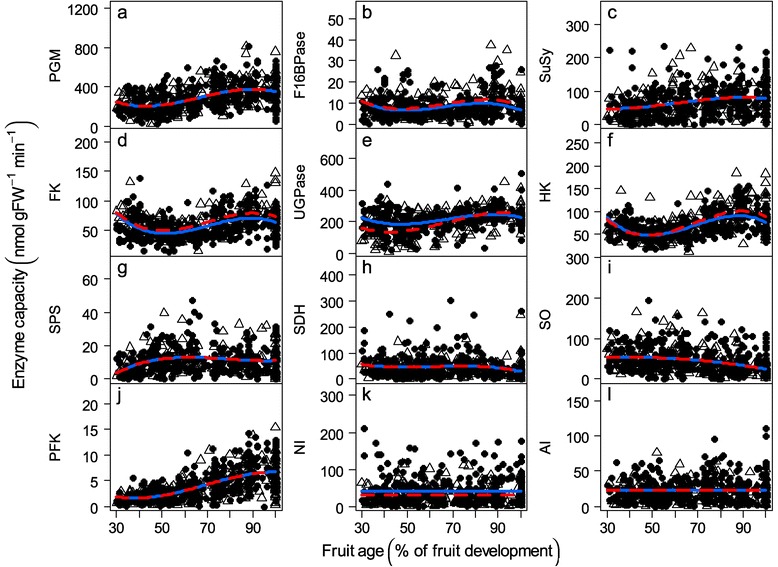
**Enzyme profiles.** Changes in the enzymatic capacities (nmol g FW ^−1^ min^−1^) during fruit development for 106 genotypes. **a)** PGM (phosphoglucomutase), **b)** F16BPase (fructose-1,6-bisphosphatase), **c)** SuSy (sucrose synthase), **d)** FK (fructokinase), **e)** UGPase (UDP-glucose pyrophosphorylase), **f)** HK (hexokinase), **g)** SPS (sucrose phosphate synthase), **h)** SDH (sorbitol dehydrogenase), **i)** SO (sorbitol oxidase), **j)** PFK (ATP-phosphofructokinase), **k)** NI (neutral invertase) and **l**) AI (acid invertase) capacities. The symbols represent the mean of the two technical replicates, open triangles represent the ‘low-fructose-to-glucose-ratio’ genotypes and closed circles the ‘standard-fructose-to-glucose-ratio’ genotypes. The lines are fitted linear models by GLMM for all of the ‘low-fructose-to-glucose-ratio’ genotypes (red dashed line) and for all of the ‘standard-fructose-to-glucose-ratio’ genotypes (blue solid line).

The enzymatic capacities were highly contrasted, with levels ranging on average from 4 to 290 nmol g FW^−1^ min^−1^ depending on the enzyme (Figure 4). The enzymes that were directly linked to hexose phosphates metabolism, especially phosphoglucomutase (PGM) and UDP-glucose pyrophosphorylase (UGPase), displayed the highest capacities (Figure 4a, e) as reported in tomato [23,31,32]. However, unlike in tomato, our study revealed that the PGM and ATP-phosphofructokinase (PFK) capacities increased during fruit growth (Figure 4a, j). The capacities of sucrose synthase (SuSy), sucrose phosphate synthase (SPS), sorbitol dehydrogenase (SDH), sorbitol oxidase (SO), neutral invertase (NI), and acid invertase (AI) (Figure 4c, g, h, i, k, l) were comparable to the data from the literature on peach [33–41], with the exception of Sun et al. [42], who reported higher and lower capacities for SPS and AI, respectively. Finally, we observed very low capacities of fructose-1,6-bisphosphatase (F16BPase) and PFK, both of which are linked to fructose-1,6-bisphosphate (F16BP) (Figure 4b, j). The temporal variations in the enzymatic capacities were also fairly small compared to those observed for the metabolite concentrations. The SuSy capacity slightly increased, fructokinase (FK) and hexokinase (HK) decreased and then increased during the second half of fruit development. F16BPase and UGPase followed the same trends to a lesser extent. SPS increased and rapidly became constant, and SO decreased at the end of growth. The capacities of the three other enzymes, SDH, NI and AI, were rather stable throughout fruit development (Figure 4h, k, l). Different studies conducted in fruits reported a rapid decrease in the capacities of the majority of the enzymes during cell division (before 50 or 60 days after bloom in peach fruit) [34,35,40]. However, in the present study, we began monitoring approximately 50–60 DAB (day after bloom) once the cell division phase was completed.

### Changes in the enzymatic capacities do not explain variations in their substrates or products

**Figure 5:**
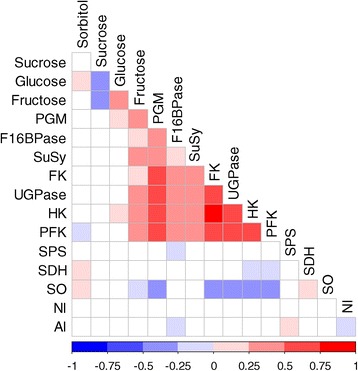
**Metabolite and enzyme correlations.** Visualization of Spearman’s correlations between the rate of metabolite production (nmol g FW^−1^ min^−1^) and the enzymatic capacities during peach fruit development for the 77 ‘standard-fructose-to-glucose-ratio’ genotypes. The square color corresponds to the correlation value as shown in the legend: blue represents a negative correlation, and red represents a positive correlation. The white squares correspond to non-significant correlations (*P* value >0.01). Abbreviations: PGM, phosphoglucomutase; F16BPase, fructose-1,6-bisphosphatase; SuSy, sucrose synthase; FK, fructokinase; UGPase, UDP-glucose pyrophosphorylase; HK, hexokinase; SPS, sucrose phosphate synthase; SDH, sorbitol dehydrogenase; SO, sorbitol oxidase; PFK, ATP-phosphofructokinase; NI, neutral invertase; AI, acid invertase.

To search for possible links between enzymatic capacities and metabolite composition, a correlation analysis was performed based on the following hypothesis: variations in the enzymatic capacities during a given period would affect the flux toward a metabolite as accumulated during this period. Therefore, the accumulation rates of metabolites were computed between two successive time points (see the Methods section) and compared using Spearman’s correlations to the average enzymatic capacities at these two time points (Figure 5). This analysis revealed a positive correlation between the enzymes involved in the metabolism of hexose phosphates (FK, HK, F16BPase, UGPase, PFK and PGM).

Considering the 15 enzyme-metabolite pairs for which the metabolite was either the direct substrate or the direct product of the enzyme, few significant correlations were found. Four positive correlations were observed between the enzymes and their direct substrate: sorbitol and SDH (0.2, *P* value <0.001), sorbitol and SO (0.17, *P* value <0.001), glucose and HK (0.15, *P* value =0.001), and fructose and FK (0.23, *P* value <0.001). The fructose accumulation rate was positively correlated to the SuSy capacity (0.25, *P* value <0.001). A single correlation was found between an enzyme and its direct product. Therefore, SuSy and FK seemed to have a greater effect on the fructose levels than AI, NI and SDH do.

### Fruit of the ‘low-fructose-to-glucose-ratio’ genotypes has a significantly lower fresh mass than fruit of the ‘standard-fructose-to-glucose-ratio’ genotypes

**Figure 6:**
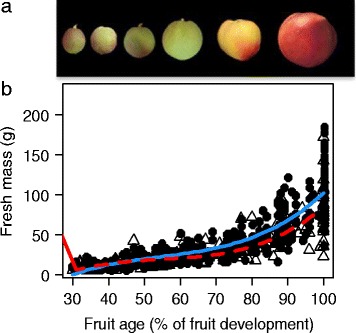
**Fruit growth. a)** Fruit harvested at different time points during development. **b)** Peach fruit growth curve in fresh mass (g) during fruit development for 106 genotypes. The symbols represent the mean of six fruits, open triangles represent the ‘low-fructose-to-glucose-ratio’ genotypes, and closed circles represent the ‘standard-fructose-to-glucose-ratio’ genotypes. The lines are fitted linear models by GLMM for all of the ‘low-fructose-to-glucose-ratio’ genotypes (red dashed line) and for all of the ‘standard-fructose-to-glucose-ratio’ genotypes (blue solid line).

For every genotype, the fruit fresh mass was monitored during fruit development (Figure 6). The ‘low-fructose-to-glucose-ratio’ genotypes had a significantly lower fresh mass than that of the ‘standard-fructose-to-glucose-ratio’ genotypes (Table 1). The difference was more pronounced during the latest developmental stage.

**Table 1:** **Variable effects on metabolites and enzymes**

	**Fructose type effect**			**Temporal effect**		
	**Chisq**	***P*** **value**		**Chisq**	***P*** **value**	
FW	42.5	6.0E-10	****	1044.4	8.3E-225	****
Glucose	17.3	1.7E-04	***	32.8	1.0E-06	****
Fructose	274.2	8.7E-60	****	383.4	1.3E-82	****
Sucrose	68.6	1.2E-15	****	457.0	1.3E-97	****
Sorbitol	11.7	3.2E-03	**	273.5	3.3E-58	****
G1P	9.7	7.6E-03	**	73.5	4.1E-15	****
G6P	3.9	1.4E-01		12.4	1.4E-02	*
F6P	6.5	3.7E-02	*	56.3	1.6E-11	****
PGM	2.7	2.5E-01		212.5	7.8E-45	****
F16BPase	6.7	3.5E-02	*	42.4	1.3E-08	****
SuSy	0.3	8.7E-01		42.7	1.2E-08	****
FK	8.9	1.1E-02	*	168.6	2.1E-35	****
UGPase	37.7	6.3E-09	****	176.2	4.7E-37	****
HK	11.0	4.1E-03	**	277.1	9.3E-59	****
SPS	1.3	5.2E-01		36.3	2.4E-07	****
SDH	2.5	2.8E-01		21.3	2.7E-04	***
SO	1.5	4.6E-01		92.3	4.2E-19	****
PFK	4.4	1.1E-01		399.8	3.1E-85	****
NI	8.5	1.4E-02	*	3.8	4.2E-01	
AI	1.1	5.6E-01		6.0	1.9E-01	

Chi square test for the fructose types and temporal effect through the 106 genotypes as obtained by a likelihood ratio test of GLMM models. ****Significant differences at *P* value <0.0001, ***Significant differences at *P* value <0.001, **Significant differences at *P* value <0.01, *Significant differences at *P* value <0.05. Abbreviations: *G1P*, Glucose-1-phosphate; *G6P*, Glucose-6-phosphate; *F6P*, Fructose-6-phosphate; *PGM*, Phosphoglucomutase; *F16BPase*, Fructose-1,6-bisphosphatase; *SuSy*, Sucrose synthase; *FK*, Fructokinase; *UGPase*, UDP-glucose pyrophosphorylase; *HK*, Hexokinase; *SPS*, Sucrose phosphate synthase; *SDH*, Sorbitol dehydrogenase; *SO*, Sorbitol oxidase; *PFK*, ATP-phosphofructokinase; *NI*, Neutral invertase; *AI*, Acid invertase.

### Striking differences in the metabolite composition associated with the fructose type

The fructose-to-glucose ratio was significantly different between the two groups of genotypes (Chisq 94, *P* value <0.001) throughout fruit growth. At maturity, there was no overlapping of the fructose-to-glucose ratios of the two fructose types. The mean, minimal and maximal values of the fructose-to-glucose ratios for the two fructose types are presented in Table 2. The fructose-to-glucose ratio was also stable between years for the two genotypes as monitored over two years (Additional file 2).

**Table 2:** **Summary of the fructose-to-glucose ratio**

**100% Development**	**Mean ± sd**	**Min**	**Max**
‘Low-fructose-to-glucose-ratio’ genotypes	0.062 ± 0.058	0.000	0.088
‘Standard-fructose-to-glucose-ratio’ genotypes	0.871 ± 0.205	0.456	1.990

The fructose-to-glucose ratio average with standard deviation and minimal (min) and maximal (max) values for the two fructose types on the final sampling date.

The two fructose types showed similar time courses for metabolite accumulation, except for sucrose, which accumulated faster in the ‘low-fructose-to-glucose-ratio’ genotypes (Figure 2c). In addition, the ‘low-fructose-to-glucose-ratio’ genotypes had significantly higher concentration of glucose, sucrose (during the second half of fruit development), sorbitol, G1P and F6P, whereas G6P was not significantly different between the two fructose types (Figures 2, 3, Table 1). The higher sucrose and glucose concentration for the ‘low-fructose-to-glucose-ratio’ genotypes resulted in a similar sweetness score between the two fructose types at maturity.

### Fruit of the ‘low-fructose-to-glucose-ratio’ genotypes have higher fructokinase and lower neutral invertase capacities

The enzymatic capacities were highly conserved between the two fructose types. Indeed, out of the twelve assayed enzymes, only five showed slight but significant differences between the two fructose types. Among these enzymes, it is interesting to note that the capacity of FK, which catalyzes the phosphorylation of fructose, was higher in the ‘low-fructose-to-glucose-ratio’ genotypes (Figure 4, Table 1). A further interesting point concerns neutral invertase (NI), which catalyzes the hydrolysis of sucrose into fructose and glucose and was lower in the ‘low-fructose-to-glucose-ratio’ genotypes (Figure 4, Table 1).

**Figure 7:**
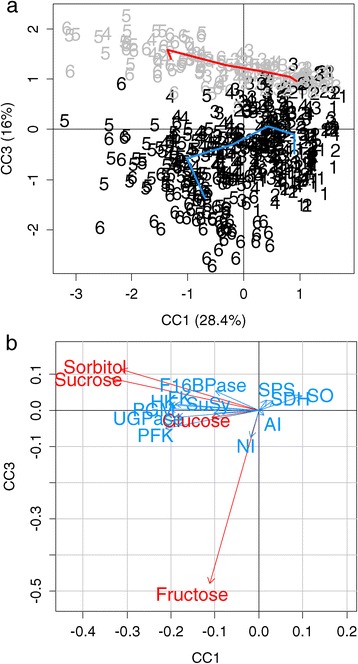
**MCOA analysis.** A multiple co-inertia analysis (MCOA) of metabolites and enzymatic capacities both expressed in nmol g FW ^−1^ min^−1^ for 106 genotypes. **“a”** represents the trajectory plots during fruit development for the first common component (CC1) and the third common component (CC3) plans. Numbers one to six correspond to values that were extrapolated from GLMM for all of the genotypes, at 40, 52, 64, 76, 88 and 100% of fruit development respectively. The grey numbers correspond to the 29 ‘low-fructose-to-glucose-ratio’ genotypes, and the black numbers correspond to the 77 ‘standard-fructose-to-glucose-ratio’ genotypes. Mean values of the fructose types at each point are linked by a line in red for the ‘low-fructose-to-glucose-ratio’ genotypes and in blue for the ‘standard-fructose-to-glucose-ratio’ genotypes. **“b”** represents the variable correlation plots that are associated with a, with metabolite variables in red and enzyme variables in blue. Abbreviations: PGM, phosphoglucomutase; F16BPase, fructose-1,6-bisphosphatase; SuSy, sucrose synthase; FK, fructokinase; UGPase, UDP-glucose pyrophosphorylase; HK, hexokinase; SPS, sucrose phosphate synthase; SDH, sorbitol dehydrogenase; SO, sorbitol oxidase; PFK, ATP-phosphofructokinase; NI, neutral invertase; AI, acid invertase.

Could these subtle differences observed in the enzymatic capacities explain the difference in the fructose concentrations between the two fructose types? To answer this question, a multiple co-inertia analysis (MCOA) was performed. This method accurately highlights trends or co-relationships in multiple datasets (Figure 7). The information that is displayed by the first common component (CC1) essentially corresponded to changes over time in the metabolite composition and enzymatic capacities (Figure 7a). The temporal trend was largely explained by sucrose and sorbitol (Figure 7b). The second common component did not allow the drawing of a pertinent feature (data not shown), whereas the third common component (CC3) was able to separate the two groups of genotypes (Figure 7a). However, the major part of the variance of this component was explained by fructose and, to a lesser extent, by sorbitol and sucrose, which emphasizes the fact that enzymatic capacities did not participate in the fructose type separation in this MCOA plan. Again, there was no obvious link between the capacities of enzymes and their direct substrates or products.

## Discussion

This study investigates sugar metabolism during peach fruit development and across significant genetic diversity, thus extending recent studies of carbon metabolism in fruit of different species [6,21,22,24] toward a full description of both the metabolites and enzymatic capacities in the progeny. The use of a large number of genotypes removes the complex effect of the genetic background, highlighting the common trends and revealing the effects of a contrasted fructose-to-glucose ratio.

### Disruption of the parallel fructose and glucose profiles in the progeny

The progeny studied here comes from an interspecific cross between a wild species (*P. davidiana*) and two commercial varieties (‘Summergrand’ and ‘Zephyr’) with contrasting phenotypic characteristics. In contrast to the varieties that bear fruit of high quality, *P. davidiana*, is characterized by small fruit with very low sugar concentrations [43]. In particular, both glucose and fructose concentrations are very low at maturity. In ‘Summergrand’ and ‘Zephyr’ fruit, glucose and fructose concentrations are similar, as usually observed in commercial fruit. In contrast, in the resulting progeny, concentrations of glucose and fructose were dissociated. Although the glucose concentration significantly increased in every genotype compared to that of *P. davidiana*, many individuals showed a very low concentration of fructose and an unbalanced fructose-to-glucose ratio. This ratio varied in a very large range (0 to 2 at maturity, Table 2), indicating that the glucose and fructose controls may be uncoupled in the progeny. This observation is consistent with the results from a QTL (quantitative trait loci) study that was previously performed on this population [44]. Accordingly, some regions of the genome control the concentrations of both glucose and fructose (linkage groups 4 and 7), whereas other regions control only glucose (linkage group 5) or fructose (linkage group 1). Interestingly, a major locus specific to the ‘low-fructose-to-glucose-ratio’ genotype (locus FRU) has also been mapped to linkage group 1 [44].

In agreement with such complex genotypic control, the ‘low-fructose-to-glucose-ratio’ genotypes do not only differ from the other genotypes at the locus controlling this character, making it difficult to grasp the mechanisms behind the ‘low-fructose-to-glucose-ratio’ genotype. The comparison between the two groups of individuals shows that the genotypes with a low fructose-to-glucose ratio have a higher concentration of other sugars (sucrose, glucose and sorbitol) and of two of the three assayed hexose phosphates (G1P and F6P). Among the studied enzymes, FK, HK and F16BPase had slightly higher capacities in these genotypes, especially during the final developmental phases. In contrast, UGPase and NI displayed a slightly lower capacity.

These particularities may have several origins. Differences between the two fructose types could arise from the systemic structure of the network controlling sugar metabolism. Changes in one or few components may indeed affect the concentration of many metabolites due to the shared control that is typical of metabolic networks. In addition, these particularities could be the result of a physical link between the genes that control these traits on the genome, indicating that these genes would participate in the observed phenotype without having any obvious link to fructose concentration. Our results show that the genotypes with a low fructose-to-glucose ratio have significantly smaller fruits than do the ‘standard-fructose-to-glucose-ratio’ genotypes. Interestingly, a fresh weight QTL was identified near locus FRU [44]. Thus, the difference in fruit weight that was observed between the two fructose types may arise from a physical connection between the two adjacent loci. Unfortunately, the low density of the genetic map and the high number of genes with unknown function preclude drawing conclusions on this point.

### High sucrose concentration, another particularity of the ‘low-fructose-to-glucose-ratio’ phenotype

The sucrose concentration was significantly higher in the ‘low-fructose-to-glucose-ratio’ genotypes during the second half of fruit development (approximately 35% higher than ‘standard-fructose-to-glucose-ratio’ genotypes at maturity). This higher sucrose concentration may explain that no difference in sweetness was found between the two fructose types.

NI was shown to have an important role on sucrose regulation in Arabidopsis [45]. The slightly lower NI capacity observed in the ‘low-fructose-to-glucose-ratio’ genotypes could lead to higher sucrose and lower hexose concentrations.

Although the lower NI capacity can account for part of the phenotype, it is not enough to explain the large increase in the sucrose concentration in the ‘low-fructose-to-glucose-ratio’ genotypes. The capacity of the other cytoplasmic and vacuolar sucrose-cleaving enzymes, SuSy and AI, appeared to be unaffected; the capacity of SPS, which is involved in sucrose re-synthesis, was also unaffected. The differences that were observed in F6P concentration could lead to a change in the re-synthesis rate of sucrose via SPS, thereby contributing to the increased sucrose concentration in the ‘low-fructose-to-glucose-ratio’ genotypes. However, the SPS flux also depends on the concentration of UDP glucose, which was not assayed in this study.

Another possibility includes cell wall invertase, another sucrose-cleaving enzyme that is localized on the apoplasm and whose capacity was not assayed in this study. Indeed a switch in the phloem-unloading mechanism has been reported to be driven by cell wall invertase during fruit development in apple and grape [46,47]. We cannot exclude that an alteration in the cell wall invertase capacity would be partly responsible for the increased sucrose concentration in the ‘low-fructose-to-glucose-ratio’ genotypes.

### High fructokinase: a candidate to explain the low fructose-to-glucose ratio

Due to the recent annotation of the peach genome [1], an exploration of the genes that are present in the locus FRU region, associated with the ‘low-fructose-to-glucose-ratio’ phenotype, was carried out. In this region, two genes encoding SuSy, which catalyzes a reaction leading to fructose (see above), have been annotated [1].

Unlike invertase, SuSy and SDH have the potential to modify the fructose-to-glucose ratio, and SDH has previously been proposed to be responsible for the regulation of the fructose concentration in peach fruit [3]. However, the data presented here do not reveal any difference in the SDH or SuSy capacities between the two fructose types, although a difference in the affinity of these two enzymes could also explain the ‘low-fructose-to-glucose-ratio’ phenotype, an option that was not investigated in this study.

It must be noted that the density of the genetic map used to detect QTLs was relatively low and that despite the quality of the annotation, many predicted genes are still unknown. Thus, in the region of the locus FRU, 39 proteins have an unknown function, allowing for many other possibilities [1].

Interestingly, a difference in the FK capacity was highlighted between the two fructose types in our study. The higher FK capacity in the ‘low-fructose-to-glucose-ratio’ genotypes would indicate a higher fructose phosphorylation flux, thus resulting in a lower fructose and a higher hexose phosphate concentration, as supported by the present data. The re-synthesis of sucrose from the hexose phosphates (via UGPase, SPS and sucrose phosphatase) would eventually result in an increase in the fructose-to-glucose ratio. The difference in the FK capacity could partially explain the excess sucrose and glucose.

Kanayama et al. [48] demonstrated the presence of two isoforms of FK with different affinities for fructose. The presence of a larger amount of the isoform with a high affinity for fructose may also explain the phenotype. FK seems to be a good candidate to explain the ‘low-fructose-to-glucose-ratio’ phenotype; however, no gene has been annotated as coding for FK in the locus FRU, and the closest gene encoding a probable fructokinase is 7 Mb away [1].

### An alternative hypothesis to concurrently explain the different features of the ‘low-fructose-to-glucose-ratio’ phenotype

Sugar metabolism is controlled by a complex network and is highly dependent on cell compartmentalization. Indeed, sucrose, glucose and fructose are stored in vacuoles; therefore, the major part of the pool of these compounds is not available as substrate for enzymes of the cytosol. This compartmentalization indeed appears to be a major regulator of sugar metabolism [49–51].

Low fructose storage could be the origin of a ‘low-fructose-to-glucose-ratio’ phenotype. In Arabidopsis leaves, the fructose concentration depends on a specific tonoplastic transporter that exports fructose out of the vacuole with a higher affinity for fructose than for glucose [52]. In this case, the glucose concentration would eventually increase within the vacuole by the hydrolysis of sucrose via AI, whereas the fructose that is concurrently produced would be exported and then rapidly metabolized in the cytoplasm. A high cytosolic fructose concentration may also have a negative feedback on the NI capacity [53], as observed in the present study.

In agreement with this hypothesis, low fructose genotypes have a significantly higher concentration in F6P (Figure 3c, Table 1), suggesting that higher fructose phosphorylation took place due to the difference in FK capacity. Higher rates of F6P synthesis could lead to higher fluxes toward glycolysis and the TCA cycle. We also found a slightly higher F16BPase capacity in these genotypes (Figure 4b, Table 1). F16BPase converts F16BP back into F6P, which potentially counteracts the flux into glycolysis and respiration. This system is highly regulated by fructose-2,6-bisphosphate, which is a strong inhibitor of F16BPase in leaves [54] and fruit [55]. A decrease in fructose-2,6-bisphosphate results in the activation of F16BPase and in higher F6P concentration [56]. This mechanism is in line with the fact that no significant difference in respiration has been found between the genotypes of the two groups (Additional file 3). This hypothesis has the merit of concurrently explaining the different features associated with the ‘low-fructose-to-glucose-ratio’ phenotype. However, although appealing, this hypothesis remains purely speculative as there is no evidence for a tonoplast transporter that is specific to fructose as expressed in peach fruit. Further work is required to confirm the existence of such a mechanism.

## Conclusions

This study, which provides a large dataset for sugars and related enzymatic capacities during peach fruit development, reveals a highly regulated system in which a major perturbation in a central compound had only slight repercussions on enzymatic capacities.

We suggest that none of the measured enzymes is able to explain the ‘low-fructose-to-glucose-ratio’ phenotype by itself. The slight variations in the observed enzymatic capacities may rather refer to an adjustment of the whole network to an external constraint imposed on the system. Following a switch in fructose metabolism that may have appeared during peach evolution, our results suggest that sugar metabolism is adjusted through slight changes in enzymatic capacities in a way that minimizes perturbations in the outputs of the pathway.

Further studies are required to explore the functional hypotheses (i.e., enzyme affinity, transporter). A modeling approach could contribute to a better understanding of the key mechanisms involved in sugar accumulation during peach fruit growth.

This study offers an overview of sugar metabolism with natural variation on peach fruit. This is the starting point for the comprehension of the mechanisms involved in sugar metabolism in fruit, providing new ways to improve fruit quality.

## Methods

### Plant material

The peach genotypes were previously studied by Quilot et al. [57] and come from a progeny obtained by two subsequent backcrosses between *Prunus davidiana* (Carr.) P1908 and *Prunus persica* (L.) Batsch ‘Summergrand’ and then ‘Zephyr.’ These plants were planted in a completely randomized design in the orchard of the INRA Research Centre of Avignon (southern France). The trees were three years old when planted in the orchard in 2001. All of the genotypes were grafted onto GF305 seedling rootstocks and were grown under normal irrigation, fertilization and pest-control conditions. All of the trees were homogeneously pruned and thinned. This study was performed on 106 different genotypes that were harvested in 2012. In addition, two of these genotypes were also studied in 2010 and 2011 (Additional file 2).

The genotypes were selected to have sufficient fruits for this experiment. Seventy-seven genotypes were considered ‘standard fructose-to-glucose ratio’ because of the balanced fructose-to-glucose ratio at maturity, which corresponds to the ratio that is found in commercial varieties, and twenty-nine genotypes were called ‘low fructose-to-glucose ratio’ due to the lower proportion of fructose compared to glucose based on their sugar composition at maturity from previous years. The fruit development period varied from 112 to 186 days after bloom depending on the genotype.

### Sample preparation

For each genotype, the maturity date was extrapolated from previous data. Maturity was reached when the fruits were no longer growing, softened and easily picked. The expected interval between the bloom and maturity dates was divided into six equal periods. The sampling points were different between the genotypes because of the different durations of fruit development. For the metabolite and enzyme assays, six fruits were collected on each sampling date. These fruits were weighed and pooled to consider the variability present on a tree. For the hexose phosphates, nine fruits were harvested at each sampling date from ten genotypes. The fruit was separated into three pools of three fruits (three biological replicates). The fruits were peeled, and the mesocarp was cut into small pieces, immediately snap frozen in liquid nitrogen and stored at −80°C. The samples were then ground in liquid nitrogen to a fine powder and stored at −80°C for future analyses. Two technical replicates were performed for each sample. The technical replicates correspond to two distinct extractions and assays.

### Extraction and measurements of metabolites

For the metabolite assays, 20-mg aliquots of powdered mesocarp were extracted as described in Gibon et al. [58]. The supernatant was used for the assay of hexoses, sorbitol, hexose phosphates.The glucose, fructose and sucrose concentrations were measured as described in Gibon et al. [58]. The sorbitol concentration was determined using 20-μl aliquots of extract, which were pipetted into microplate wells containing 0.01 mmol Tricine/KOH pH 9.5 and 0.1 nmol NAD^+^ in a final volume of 100 μl. After reading the absorbance at 340 nm until stability, 0.1 U of sorbitol dehydrogenase was added to each well, and the absorbance was read until stability. G1P, G6P and F6P were analyzed as described in Gibon et al. [26].

### Extraction and measurements of enzymatic capacities

For the enzymatic capacity assays, 20-mg aliquots of powdered mesocarp were extracted as described in Gibon et al. [58]. The extracts used for the determination of SPS, AI, NI, SDH, SuSy were desalted with a PD MultiTrap G25 column (GE Healthcare Life Sciences). PFK, F16BPase, HK, FK and AI were analyzed as described in Gibon et al. [58]. The PGM was determined as described in Manjunath et al. [59]. UGPase was measured as described in Biais et al. [23]. SPS and SuSy were assessed as in Lunn et al. [60]. NI was determined using 5-μl aliquots of extract that were pipetted directly into a microplate, followed by 95 μl of assay mix containing 1.25 μmol HEPES/KOH pH 7.5, 200 nmol MgCl_2_, 200 nmol ATP, 200 nmol NADP^+^, 0.0175 U of glucose-6-phosphate dehydrogenase, 0.525 U of phosphoglucose isomerase, 0.9 U of hexokinase, and 0 (blank) or 2 μmol (maximal activity) sucrose. The absorbance was read at 340 nm and 25°C until the rates were close to 0 in the blanks or stabilized in the wells in which sucrose was added. SDH and SO were assessed using 10-μl aliquots of extract, which were pipetted into a microplate containing, in each well, 10 μmol Tris pH 9.5, 300 nmol NAD^+^ (only for SDH), 100 nmol thiazolyl blue tetrazolium bromide (MTT), 0 (blank) or 45 μmol (maximal activity) sorbitol and 20 nmol phenazine ethosulfate. The absorbance was read at 340 nm until the rates were stabilized.

### Statistical analysis

Statistical analyses of the mean of technical replicates were performed using R software (R Development Core Team 2006). The time courses (fruit age) are represented according to the percentage of fruit development, where 0% corresponds to the bloom date and 100% to fruit maturity.

To detect significant differences between the two fructose types and the effect of the time course profile, a generalized linear mixed-effects model (GLMM) was used as described in Bugaud et al. [61]. The lmer function in the ‘lmer4’ library was used. A complete model including the fructose type effect, the percentage of fruit development effect and their interactions, as well as quadratic and cubic terms for the percentage of fruit development (df: 8), was compared to a model including the percentage of fruit development, quadratic and cubic terms for the percentage of fruit development (df: 6) and to another model including only the fructose type (df: 4). For these three models, a random effect was added depending on the genotype. A comparison of the three models was performed with a likelihood ratio test using the ANOVA function in the R software.

A multiple co-inertia analysis (MCOA) (R software, ‘ade4’ library) was performed for both the metabolite and enzyme data as described in Mazerolles et al. [62]. For every genotype, a GLMM was used to predict the data at 40, 52, 64, 76, 88 and 100% of fruit development to compare all of the genotypes at an equivalent percentage of fruit development.

To investigate the links between the metabolite concentrations and the enzymatic capacities, the accumulation rates of the metabolites were compared to the enzymatic capacities. The accumulation rates of the metabolites were computed between two successive time points: the difference in the metabolite concentration between the two sampling points was divided by the number of minutes that had passed.

The normality of the data was tested with the Shapiro-Wilk normality test. Given that the data did not follow a normal distribution, the Spearman method was used for the correlation analyses. Spearman’s correlations were calculated and plotted using the ‘corrplot’ library (R software). Only significant correlations (*P* value <0.01) were plotted.

The sweetness was calculated for all genotypes at maturity according to the perceived sweetness scores cited by Cantin et al. [20] for fructose (1.75), glucose (0.75) and sucrose (1).

## Additional files

Additional file 1: Table S1. A summary of the published studies. Review of the studies that have been performed on peach fruit at maturity or during fruit development and that described the sugar concentrations and enzymatic capacities. Additional file 2: Figure S1. Developmental profiles of metabolites and enzymes in two genotypes during two years. Changes in metabolite concentrations in mg g FW ^−1^ and enzymatic capacities (nmol g FW ^−1^ min^−1^) during fruit development (DAB, day after bloom) for two genotypes (one ‘standard-fructose-to-glucose-ratio’ genotype in blue and one ‘low-fructose-to-glucose-ratio’ genotype in red) and two years (2010: solid line, 2011 dashed line). Symbols represent the mean and standard deviation of three biological replicates of three fruits each and lines are fitted linear models by GLMM. Additional file 3: Figure S2. CO_2_ and respiratory quotient. Changes in the amount of CO_2_ that was produced per minute per kilogram of fruit (a) and respiratory quotient (b) represented by the ratio of CO_2_ that was produced to O_2_ that was consumed during growth (DAB, day after bloom). The measurements were performed during three stages of fruit development and for one ‘low-fructose-to-glucose-ratio’ genotype in white boxes as connected by a dashed line and for one ‘standard-fructose-to-glucose-ratio’ genotype in gray boxes and connected by a solid black line. The genotypes that were used here were the same as those represented in Additional file 2. The measurements were carried out on fruits that were attached to the tree and enclosed in hermetic boxes. The same fruits were monitored on the three dates. Five fruits were measured on each date and for each genotype.

## Abbreviations

AI: Acid invertase
DAB: Day after bloom
F16BPase: Fructose-1,6-bisphosphatase
F16BP: Fructose 1,6 bisphosphate
F6P: Fructose-6-phosphate
FK: Fructokinase
FW: Fresh weight
G1P: Glucose-1-phosphate
G6P: Glucose-6-phosphate
GLMM: Generalized linear mixed-effects model
HK: Hexokinase
MCOA: Multiple co-inertia analysis
NI: Neutral invertase
PFK: ATP-phosphofructokinase
PFP: PPi-phosphofructokinase
PGI: Phosphoglucose isomerase
PGM: Phosphoglucomutase
QTL: Quantitative trait loci
SDH: Sorbitol dehydrogenase
SO: Sorbitol oxidase
SPP: Sucrose-phosphate phosphatase
SPS: Sucrose phosphate synthase
SuSy: Sucrose synthase
UGPase: UDP-glucose pyrophosphorylase
