# Supplementary material for: Profiling sugar metabolism during fruit development in a peach progeny with different fructose-to-glucose ratios
Source: BMC Plant Biol. 2014 Nov 25;14:336. doi: 10.1186/s12870-014-0336-x (PMC4247632; doi:10.1186/s12870-014-0336-x)
Supplement: Additional file 1: Table S1. — A summary of the published studies. Review of the studies that have been performed on peach fruit at maturity or during fruit development and that described the sugar concentrations and enzymatic capacities. [file 12870_2014_336_MOESM1_ESM.pdf]

Additional File 1: Table S1. A summary of the published studies. Review of the studies that have been performed on peach fruit at maturity or during fruit development and that described the sugar concentrations and enzymatic capacities.

	Maturity	Fruit development
Sucrose	[1-13]	[14-31]
Fructose	[1-12]	[14, 15, 17-31]
Glucose	[1-12]	[14, 15, 17-31]
Sorbitol	[1-11, 13]	[14-29]
F6P		[14, 19]
G6P		[14]
G1P		
UDP glucose		
Sucrose 6P		
AI	[2, 8]	[15-19, 21, 23, 24, 26, 28, 29]
NI	[2, 8]	[16-19, 21, 26, 28]
SuSy	[8]	[15-18, 21, 23, 24, 26, 28, 29]
SDH	[8]	[14, 16-18, 21, 32]
SO	[8]	[15-17, 21, 23, 29]
SPS	[8]	[15, 23, 24, 26, 28, 29]
FK	[2]	[14]
UGPase	[2]	[24]
HK	[2]	
PFK	[2]	
PFP	[2]	
PGI		[14]
F16BPase		[14]
PGM		
SPP		

1. Alcobendas R, Mirás-Avalos J, Alarcón JJ, Nicolás E: **Effects of irrigation and fruit position on size, colour, firmness and sugar contents of fruits in a mid-late maturing peach cultivar**. *Sci. Hort.* 2013, **164**:340-347.
2. Borsani J, Budde C, Porrini L, Lauxmann M, Lombardo V, Murray R, Andreo C, Drincovich M, Lara M: **Carbon metabolism of peach fruit after harvest: changes in enzymes involved in organic acid and sugar level modifications**. *J. Exp. Bot.* 2009, **60**(6):1823-1860.
3. Brooks SJ, Moore JN, Murphy JB: **Quantitative and qualitative changes in sugar content of peach genotypes *Prunus persica* (L.) Batsch** *J. Am. Soc. Hort. Sci.* 1993, **118**(1):97-100.
4. Byrne DH, Nikolic AN, Burns EE: **Variability in sugars, acids, firmness, and color characteristics of 12 peach genotypes**. *J. Am. Soc. Hort. Sci.* 1991, **116**(6):1004-1006.
5. Cantín C, Gogorcena Y, Moreno MA: **Analysis of phenotypic variation of sugar profile in different peach and nectarine [*Prunus persica* (L.) Batsch] breeding progenies**. *J. Sci. Food Agric.* 2009, **89**:1909-1917.

6. Moing A, Poessel JL, Svanella-Dumas L, Loonis M, Kervella J: **Biochemical basis of low fruit quality of *Prunus davidiana*, a pest and disease resistance donor for peach breeding**. *J. Am. Soc. Hort. Sci.* 2003, **128**(1):55-62.
7. Orazem P, Stampar F, Hudina M: **Quality analysis of 'Redhaven' peach fruit grafted on 11 rootstocks of different genetic origin in a replant soil**. *Food Chem.* 2011, **124**:1691-1698.
8. Sun Z, Li Y, Zhou J, Zhu S-H: **Effects of exogenous nitric oxide on contents of soluble sugars and related enzyme activities in 'Feicheng' peach fruit**. *J. Sci. Food Agric.* 2011, **91**:1795-2595.
9. Wu B, Quilot B, Kervella J, Génard M, Li S: **Analysis of genotypic variation of sugar and acid contents in peaches and nectarines through the Principle Component Analysis**. *Euphytica* 2003, **132**:375-759.
10. Dirlewanger E, Moing A, Rothan C, Svanella L, Pronier V, Guye A, Plomion C, Monet R: **Mapping QTLs controlling fruit quality in peach (Prunus persica (L.) Batsch)**. *Theor. Appl. Genet.* 1999, **98**:18-49.
11. Font i Forcada C, Gogorcena Y, Moreno MA: **Fruit sugar profile and antioxidants of peach and nectarine cultivars on almond×peach hybrid rootstocks**. *Sci. Hort.* 2013, **164**:563-572.
12. Esti M, Messia M, Sinesio F, Nicotra A, Conte L, La Notte E, Palleschi G: **Quality evaluation of peaches and nectarines by electrochemical and multivariate analyses: relationships between analytical measurements and sensory attributes**. *Food Chem.* 1997, **60**(4):659-666.
13. Colaric M, Veberic R, Stampar F, Hudina M: **Evaluation of peach and nectarine fruit quality and correlations between sensory and chemical attributes**. *J. Sci. Food Agric.* 2005, **85**:2611-2616.
14. Kanayama Y, Kogawa M, Yamaguchi M, Kanahama K: **Fructose content and the activity of fructose - related enzymes in the fruit of eating - quality peach cultivars and native - type peach cultivars**. *J. Jpn.Soc. Hortic. Sci* 2005, **74**(6):431-436.
15. Kobashi K, Gemma H, Iwahori S: **Sugar accumulation in peach fruit as affected by abscisic acid (ABA) treatment in relation to some sugar metabolizing enzymes**. *J. Jpn.Soc. Hortic. Sci* 1999, **68**(3):465-470.
16. Lo Bianco R, Rieger M: **Roles of sorbitol and sucrose in growth and respiration of 'Encore' peaches at the three developmental stages**. *J. Am. Soc. Hort. Sci.* 2002, **127**(2):297-302.
17. Lo Bianco R, Rieger M: **Partitioning of sorbitol and sucrose catabolism within peach fruit**. *J. Am. Soc. Hort. Sci.* 2002, **127**(1):115-121.
18. Lo Bianco R, Rieger M, Sung SJS: **Carbohydrate metabolism of vegetative and reproductive sinks in the late-maturing peach cultivar 'Encore'**. *Tree Physiol.* 1999, **19**(2):103-109.
19. Lombardo V, Osorio S, Borsani J, Lauxmann M, Bustamante C, Budde C, Andreo C, Lara M, Fernie A, Drincovich M: **Metabolic profiling during peach fruit development and ripening reveals the metabolic networks that underpin each developmental stage**. *Plant Physiol.* 2011, **157**:1696-2406.
20. Moing A, Svanella L, Rolin D, Gaudillere M, Gaudillere JP, Monet R: **Compositional changes during the fruit development of two peach cultivars differing in juice acidity**. *J. Am. Soc. Hort. Sci.* 1998, **123**(5):770-775.
21. Morandi B, Corelli Grappadelli L, Rieger M, Lo Bianco R: **Carbohydrate availability affects growth and metabolism in peach fruit**. *Physiol. Plant.* 2008, **133**:229-270.
22. Moriguchi T, Ishizawa Y, Sanada T: **Differences in sugar composition in *Prunus persica* fruit and classification by the principal component analysis**. *J. Jpn.Soc. Hortic. Sci* 1990, **59**(2):307-312.
23. Moriguchi T, Sanada T, Yamaki S: **Seasonal fluctuations of some enzymes relating to sucrose and sorbitol metabolism in peach fruit**. *J. Am. Soc. Hort. Sci.* 1990, **115**(2):278-281.
24. Moriguchi T, Ishizawa Y, Sanada T, Teramoto S, Yamaki S: **Role of sucrose synthase and other related enzymes in sucrose accumulation in peach fruit** *J. Jpn.Soc. Hortic. Sci* 1991, **60**(3):531-538.

25. Pavel EW, Dejong TM: **Relative growth-rate and its relationship to compositional changes of nonstructural carbohydrates in the mesocarp of developing peach fruits**. *J. Am. Soc. Hort. Sci.* 1993, **118**(4):503-508.
26. Vizzotto G, Pinton R, Varanini Z, Costa G: **Sucrose accumulation in developing peach fruit**. *Physiol. Plant.* 1996, **96**(2):225-230.
27. Wu B, Quilot B, Génard M, Kervella J, Li S: **Changes in sugar and organic acid concentrations during fruit maturation in peaches, *P. davidiana* and hybrids as analyzed by principal component analysis**. *Sci. Hort.* 2005, **103**:429-868.
28. Zhang C, Shen Z, Zhang Y, Han J, Ma R, Korir NK, Yu M: **Cloning and expression of genes related to the sucrose-metabolizing enzymes and carbohydrate changes in peach**. *Acta Physiol. Plant.* 2013, **35**:589-602.
29. Kobashi K, Gemma H, Iwahori S: **Abscisic acid content and sugar metabolism of peaches grown under water stress**. *J. Am. Soc. Hort. Sci.* 2000, **125**(4):425-428.
30. Etienne C, Moing A, Dirlewanger E, Raymond P, Monet R, Rothan C: **Isolation and characterization of six peach cDNAs encoding key proteins in organic acid metabolism and solute accumulation: involvement in regulating peach fruit acidity**. *Physiol. Plant.* 2002, **114**:259-529.
31. Nonis A, Ruperti B, Falchi R, Casatta E: **Differential expression and regulation of a neutral invertase encoding gene from peach (*Prunus persica*): evidence for a role in fruit development**. *Physiol. Plant.* 2007, **129**:436-446.
32. Yamada K, Niwa N, Shiratake K, Yamaki S: **cDNA cloning of NAD-dependent sorbitol dehydrogenase from peach fruit and its expression during fruit development**. *J. Horticult. Sci. Biotechnol.* 2001, **76**(5):581-587.
