# Supplementary material for: Profiling sugar metabolism during fruit development in a peach progeny with different fructose-to-glucose ratios
Source: BMC Plant Biol. 2014 Nov 25;14:336. doi: 10.1186/s12870-014-0336-x (PMC4247632; doi:10.1186/s12870-014-0336-x)
Supplement: Additional file 2: Figure S1. — Developmental profiles of metabolites and enzymes in two genotypes during two years. Changes in metabolite concentrations in mg g FW −1 and enzymatic capacities (nmol g FW −1 min−1) during fruit development (DAB, day after bloom) for two genotypes (one ‘standard-fructose-to-glucose-ratio’ genotype in blue and one ‘low-fructose-to-glucose-ratio’ genotype in red) and two years (2010: solid line, 2011 dashed line). Symbols represent the mean and standard deviation of three biological replicates of three fruits each and lines are fitted linear models by GLMM. [file 12870_2014_336_MOESM2_ESM.pdf]

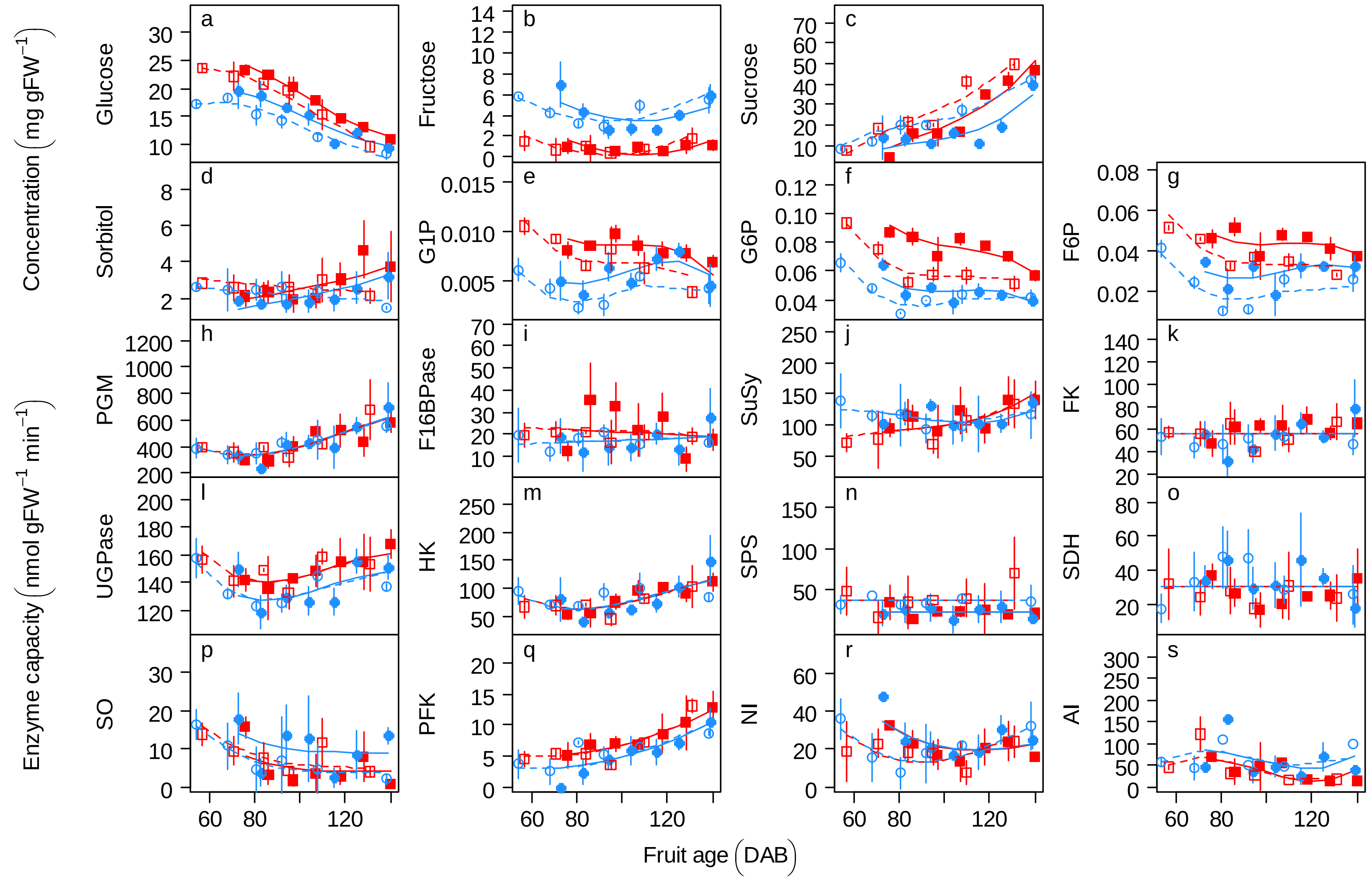

Additional file 2: Figure S1. Developmental profiles of metabolites and enzymes in two genotypes during two years. Changes in metabolite concentrations in mg g FW $^{-1}$ and enzymatic capacities (nmol g FW $^{-1}$ min^{-1}) during fruit development (DAB, day after bloom) for two genotypes (one 'standard-fructose-to-glucose-ratio' genotype in blue and one 'low-fructose-to-glucose-ratio' genotype in red) and two years (2010: solid line, 2011 dashed line). Symbols represent the mean and standard deviation of three biological replicates of three fruits each and lines are fitted linear models by GLMM.
