# Supplementary material for: Profiling sugar metabolism during fruit development in a peach progeny with different fructose-to-glucose ratios
Source: BMC Plant Biol. 2014 Nov 25;14:336. doi: 10.1186/s12870-014-0336-x (PMC4247632; doi:10.1186/s12870-014-0336-x)
Supplement: Additional file 3: Figure S2. — CO2 and respiratory quotient. Changes in the amount of CO2 that was produced per minute per kilogram of fruit (a) and respiratory quotient (b) represented by the ratio of CO2 that was produced to O2 that was consumed during growth (DAB, day after bloom). The measurements were performed during three stages of fruit development and for one ‘low-fructose-to-glucose-ratio’ genotype in white boxes as connected by a dashed line and for one ‘standard-fructose-to-glucose-ratio’ genotype in gray boxes and connected by a solid black line. The genotypes that were used here were the same as those represented in Additional file 2. The measurements were carried out on fruits that were attached to the tree and enclosed in hermetic boxes. The same fruits were monitored on the three dates. Five fruits were measured on each date and for each genotype. [file 12870_2014_336_MOESM3_ESM.pdf]

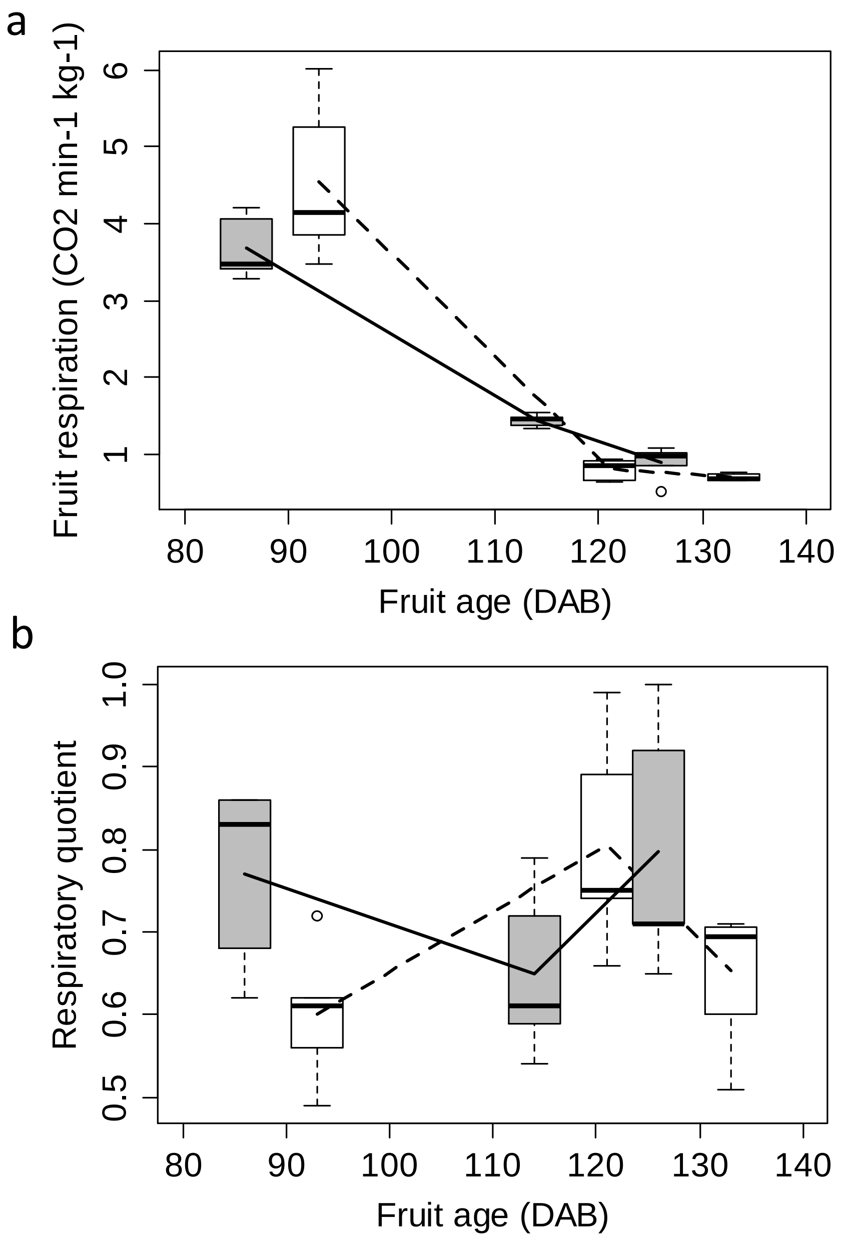

Additional File 3: Figure S2. CO_2 and respiratory quotient. Changes in the amount of CO_2 that was produced per minute per kilogram of fruit (a) and respiratory quotient (b) represented by the ratio of CO_2 that was produced to O_2 that was consumed during growth (DAB, day after bloom). The measurements were performed during three stages of fruit development and for one 'low-fructose-to-glucose-ratio' genotype in white boxes as connected by a dashed line and for one 'standard-fructose-to-glucose-ratio' genotype in gray boxes and connected by a solid black line. The genotypes that were used here were the same as those represented in Additional File 2. The measurements were carried out on fruits that were attached to the tree and enclosed in hermetic boxes. The same fruits were monitored on the three dates. Five fruits were measured on each date and for each genotype.
